# Evaluating Motivational Interviewing and Habit Formation to Enhance the Effect of Activity Trackers on Healthy Adults’ Activity Levels: Randomized Intervention

**DOI:** 10.2196/10988

**Published:** 2019-02-14

**Authors:** Laura D Ellingson, Jeni E Lansing, Kathryn J DeShaw, Karissa L Peyer, Yang Bai, Maria Perez, L Alison Phillips, Gregory J Welk

**Affiliations:** 1 Department of Kinesiology Iowa State University Ames, IA United States; 2 Department of Health and Human Performance University of Tennessee-Chatanooga Chattanooga, TN United States; 3 Deparment of Rehabilitation and Movement Science University of Vermont Burlington, VT United States; 4 Department of Psychology Iowa State University Ames, IA United States

**Keywords:** activity tracker, habit, mHealth, motivational interviewing, mobile phone, physical activity, wearable electronic devices

## Abstract

**Background:**

While widely used and endorsed, there is limited evidence supporting the benefits of activity trackers for increasing physical activity; these devices may be more effective when combined with additional strategies that promote sustained behavior change like motivational interviewing (MI) and habit development.

**Objective:**

This study aims to determine the utility of wearable activity trackers alone or in combination with these behavior change strategies for promoting improvements in active and sedentary behaviors.

**Methods:**

A sample of 91 adults (48/91 female, 53%) was randomized to receive a Fitbit Charge alone or in combination with MI and habit education for 12 weeks. Active and sedentary behaviors were assessed pre and post using research-grade activity monitors (ActiGraph and activPAL), and the development of habits surrounding the use of the trackers was assessed postintervention with the Self-Reported Habit Index. During the intervention, Fitbit wear time and activity levels were monitored with the activity trackers. Linear regression analyses were used to determine the influence of the trial on outcomes of physical activity and sedentary time. The influence of habits was examined using correlation coefficients relating habits of tracker use (wearing the tracker and checking data on the tracker and associated app) to Fitbit wear time and activity levels during the intervention and at follow-up.

**Results:**

Regression analyses revealed no significant differences by group in any of the primary outcomes (all *P*>.05). However, personal characteristics, including lower baseline activity levels (beta=–.49, *P*=.01) and lack of previous experience with pedometers (beta=–.23, *P*=.03) were predictive of greater improvements in moderate and vigorous physical activity. Furthermore, for individuals with higher activity levels at the baseline, MI and habit education were more effective for maintaining these activity levels when compared with receiving a Fitbit alone (eg, small increase of ~48 steps/day, *d*=0.01, vs large decrease of ~1830 steps/day, *d*=0.95). Finally, habit development was significantly related to steps/day during (*r*=.30, *P*=.004) and following the intervention (*r*=.27, *P*=.03).

**Conclusions:**

This study suggests that activity trackers may have beneficial effects on physical activity in healthy adults, but benefits vary based on individual factors. Furthermore, this study highlights the importance of habit development surrounding the wear and use of activity trackers and the associated software to promote increases in physical activity.

**Trial Registration:**

ClinicalTrials.gov NCT03837366; https://clinicaltrials.gov/ct2/show/NCT03837366

## Introduction

Wearable technology remains popular [1], with industry experts projecting continued growth of the consumer sector [2,3]. However, despite widespread use, the utility of wearable activity trackers for improving physical activity (PA) is equivocal as highlighted in several reviews [4-6]. Thus, an important behavioral consideration is to determine how to optimally use the feedback from these monitors to most effectively facilitate behavior change.

Reportedly, activity trackers may be more effective when combined with additional behavior change strategies [7]; several studies have explored this possibility by testing the added benefits of education-based counseling and goal setting [8], as well as by integration with short message service text messaging and incentives through mobile health apps [9-11]. However, these strategies have not markedly enhanced the effectiveness of trackers. While a variety of additional strategies may be able to address this need, a recent review [12] concluded that monitors are more likely to be effective if they are accompanied by coaching or counseling from personnel with expertise in promoting behavior change.

This study aims to evaluate this recommendation by assessing the utility of a low-dose health coaching intervention to enhance outcomes associated with the use of wearable trackers. A low-dose coaching format was chosen as this would be more cost-effective and, thus, more suitable for broader translation through mobile health apps. The health coaching was based on the principles of motivational interviewing (MI). The use of MI offers advantages for clinically based health coaching, as it is designed to help build intrinsic motivation for behavior change [13]. In addition, MI has been widely used to positively influence behavior with notable apps for PA in several large intervention trials [14,15] and has been shown to improve adherence to and retention of health behaviors when combined with other behavior change strategies [16]. A novel aspect of our evaluation is in assessing whether MI-based coaching can promote more effective behavior change, incorporating the use of activity trackers. We hypothesized that those receiving health coaching along with their tracker would have greater improvements in PA and sedentary behavior than those receiving the tracker alone.

In concert with the MI-based approach, we also used contemporary theories of habit formation to promote and evaluate participants’ adoption and use of the trackers and the relationship of these behaviors to PA and sedentary time. A habit has been defined as “a goal-directed sequence of actions that becomes automatic in response to learned, contextual cues” [17]. Though evidence has demonstrated that the development of habits can be involved in adoption and adherence to PA behaviors [18-21], studies, to date, have not specifically used habit theory to understand or promote PA along with activity tracker usage. The development of habits surrounding the tracker and associated app, such as regular wear of the monitor and checking data at a time where PA could be performed (eg, during the lunch hour), could lead to more consistent use and promote sustained improvements in activity levels.

Existing theory and empirical findings from observational studies showed that behavior change occurs in 2 distinct action phases—a motivational phase, which results in the formation of a behavioral goal or intention, and a volitional phase, which results in the enactment of behavior once a goal or intention is formed [22-25]. MI in combination with habit formation may enhance both initial motivation and the completion of the volitional phase of behavioral action (ie, carrying through with one’s intentions, over time). Thus, as a secondary hypothesis, we anticipated that individuals receiving health coaching would establish stronger habits than those receiving an activity tracker alone and that greater habit development would be associated with the more consistent use of the tracker and more positive outcomes. The systematic evaluation of strategies to enhance the utilization of wearable trackers is an essential step in the more effective use of these devices in behavioral research.

## Methods

### Participants

All procedures were approved by the relevant Institutional Review Board. Participants were recruited from the campus community using electronic mailing lists. The inclusion criteria were as follows: aged 24-65 years; regular access to a computer or smartphone; and a willingness to wear an activity tracker for the study duration. The exclusion criteria were we as follows: current use of an activity tracker; the presence of health conditions that prevented safe engagement in PA; current participation in a structured exercise program; or self-reported activity levels sufficient to meet the aerobic component of PA guidelines of 150 minutes of moderate or 75 minutes of vigorous activity per week.

### Procedures

**Figure 1:**
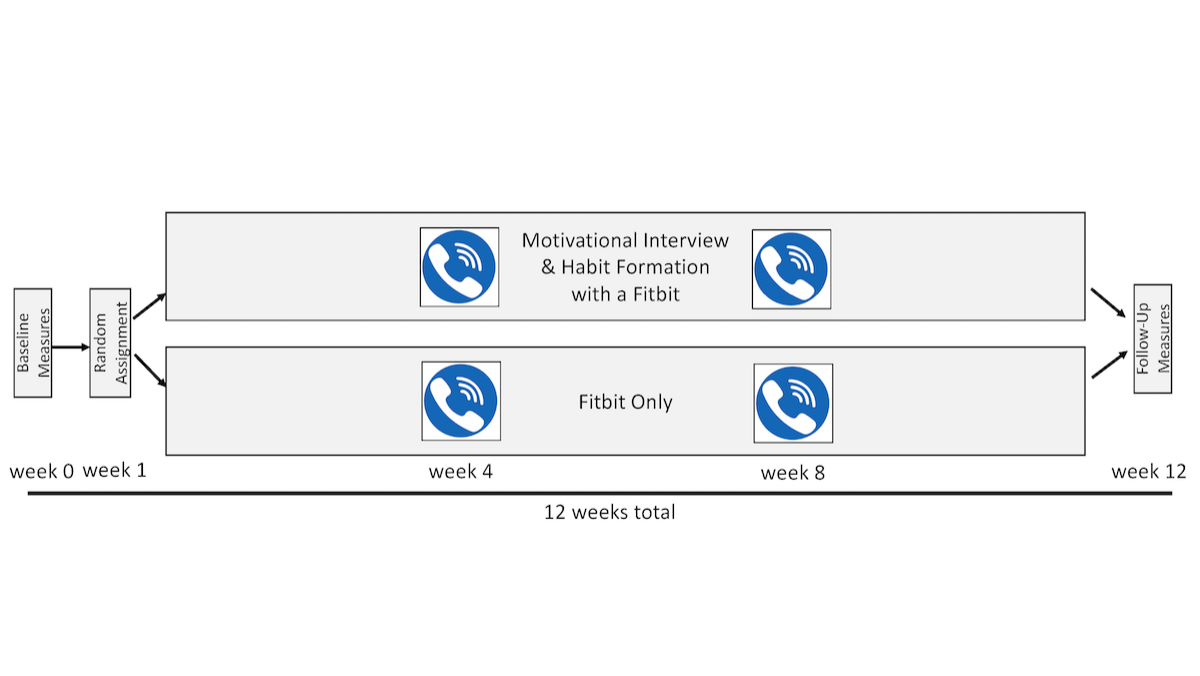
Timeline of study procedures.

This study was designed as a feasibility trial [26] to refine methods prior to implementation in larger clinical trials. The protocol involved 3 laboratory visits and 2 phone calls over 3 months (Figure 1). Data were collected in 5 cohorts, ranging from 15 to 20 participants each. The first 3 began in summer, and the fourth and fifth began in the fall. No cohorts started or finished within 1 week of a major holiday. Prior to data collection, participants read and signed the informed consent document and completed the Physical Activity Readiness Questionnaire [27] to assess eligibility. Participants then completed a demographic questionnaire, and the International Physical Activity Questionnaire [28] was given in interview format to further determine eligibility. Participants who self-reported being sufficiently active to meet guidelines (n=2) were excluded from remaining study procedures.

To characterize the sample, blood pressure and heart rate were assessed with an automated blood pressure monitor (Omron HEM712C; Omron Healthcare, Inc, Hoffman Estates, IL, USA).

While height was measured using a standard stadiometer, weight and body composition were estimated using bioelectrical impedance (InBody 720; InBody, Cerritos, CA, USA). Two research-grade activity monitors (described below) were then provided for participants to wear for 1 week to obtain baseline measures of PA and sedentary behaviors. Participants were verbally encouraged to behave as usual during this baseline assessment, and visits were rescheduled if participants indicated that the week would likely be unrepresentative of their typical behavior patterns owing to travel, illness, or other obligations.

Following the baseline monitoring period, participants were randomly assigned to receive a Fitbit alone (FB) or in combination with MI and habit education provided by a trained health coach (FB+). The FB group was intended to be comparable to a real-world setting wherein individuals purchase and utilize activity trackers on their own. Regardless of group assignment, all participants received a Fitbit Charge wrist-worn activity monitor and were instructed to use it at their discretion for the duration of the 3-month intervention. The study staff assisted participants in setting up a Web-based Fitbit account and demonstrated features of the Fitbit itself and the associated computer software and app (eg, logging food, dashboard displays, and alarms). In addition, participants assigned to the FB+ group discussed their self-determined goals regarding PA and principles of habit formation with a trained staff member. Habit education included a brief definition of habits and their relevance for sustained behavior change, followed by working with participants to determine salient cues to remember to wear their Fitbit and regularly check their data on the Fitbit itself and through the app at a time when PA was feasible. During the 3-month intervention, behaviors were monitored through Fitabase software (Small Steps, Inc; San Diego, CA, USA) as a measure of implementation, including metrics for Fitbit wear time and use and to track PA behaviors (eg, steps/day) throughout the intervention.

During the fourth and eighth weeks of the study, all participants were contacted by phone for a brief (~10-30 minute) conversation regarding their experience using the Fitbit; this included likes, dislikes, and any technical difficulties they were experiencing. In addition, individuals in the FB+ group revisited their self-selected PA goals and motivation for change. After 12 weeks, during which participants still had access to their Fitbits, the same research-grade PA monitors were distributed to assess behavior change. Participants then returned to the lab for their final visit, repeating all baseline assessments and completing a measure of habit strength surrounding the use of the Fitbit and associated software (described below).

### Measures

#### Evaluation of Physical Activity and Sedentary Time

Pre- and postintervention, PA and sedentary behaviors were assessed objectively using ActiGraph GT3X+ (ActiGraph, LLC, Fort Walton Beach, FL, USA) and activPAL3 (Physical Activity Technologies, Glasgow, UK) activity monitors. Participants were instructed to place the ActiGraph on the hip using the elastic belt and the activPAL on the midline of the thigh on either leg. For both monitors, participants were instructed to wear them during all waking hours, except when bathing or swimming. To accompany the monitors, participants were given a log sheet and asked to record time on or off for each monitor and waking hours (eg, sleep and wake times).

The ActiGraph and activPAL data were initially processed using proprietary software (ActiLife 6.13.3 and Performance Analysis of Logs Analysis 7.2.32) with outputs at the 1-second epoch. These data were then integrated through their timestamps and further analyzed using the Sojourns Including Posture method [29]. The Sojourns Including Posture method has been shown to provide a high-level validity for the full range of behaviors from sedentary to vigorous in comparison to direct observation and indirect calorimetry [29]. The output from this processing method was used to calculate the primary outcomes for the study, including average steps, minutes of moderate-to-vigorous intensity PA (MVPA; total and in 10-minute bouts) and sedentary time (total and in 30-minute bouts) per day. Change scores for these metrics were calculated by subtracting baseline scores from follow-up scores, and percent changes were then calculated using the following formula: [(follow-up–baseline)/baseline]×100.

#### Evaluation of Habit Formation

The habit development was assessed postintervention using the Automaticity Subscale from the Self-Reported Habit Index (SRHI) [30]. The SRHI was designed to be flexible such that it can be used with a variety of potentially habitual behaviors to fit study needs and has been shown to predict PA behaviors [31]. The Automaticity Subscale includes 4 of the 12 items from the full questionnaire and has been validated as a measure of habitual behavior [32]; each item is scored on a 5-point Likert scale with anchors ranging from strongly agree to strongly disagree. Behaviors in question are used as the stems of sentences that are followed by the 4 automaticity-related sentence endings. The behaviors assessed in this study were wearing the Fitbit, checking data on the Fitbit itself, and checking data on the Fitbit software or app. Thus, participants were asked to indicate the extent to which they agreed with statements like “Wearing my Fitbit is something I do without thinking” and “Using the Fitbit mobile app is something I start doing without realizing I’m doing it.” Scores on this measure are summed across the 4 items to create an automaticity index for each behavior. In this study, each of the 3 behaviors noted above was examined separately and a habit automaticity total was also created by summing the totals from each of the separate behaviors.

#### Physical Activity Behaviors During the Intervention

Fitbit wear, as well as activity levels, were monitored during the study using the Fitabase software mentioned above. The data from Fitabase were downloaded in 1-minute epochs and summarized to determine the frequency for wearing the tracker, as well as accumulation of daily steps and minutes of MVPA averaged over each week of the intervention. A valid monitor wear day was defined as accumulating steps during, at least, 10 hours/day, indicating that the monitor was being worn. Days on which the Fitbit was worn for <10 hours were excluded from analyses. Thus, metrics of interest (eg, average steps/day) were calculated using only days with sufficient wear time. Participants’ wear time, daily steps, and minutes of MVPA were tracked throughout the 12 weeks to enable indicators of habit to be directly related to the objective data.

### Statistical Analyses

Descriptive statistics were used to characterize the sample with regards to demographic variables, as well as baseline PA and sedentary behaviors. Group differences at baseline were analyzed using chi-square tests for categorical variables and independent-samples *t* tests for continuous variables. In addition, data from Fitabase were examined descriptively to assess behavior and use of the monitors during each week of the study. Data were then compared between groups using independent- samples *t* tests for each week of the intervention.

To examine the influence of the MI-based health coaching on outcomes related to active and sedentary behaviors, we first descriptively compared within- and between-groups changes using effect size calculations (Cohen *d*). To examine the influence of baseline levels of activity on the effects of the FB or in combination with MI, the sample was also further subdivided into high and low active with 7500 steps/day at the baseline, serving as the cutoff point based on the established range for being considered “active” from Tudor-Locke et al [33]. Outcomes for steps, MVPA, and sedentary time were again compared within these subgroups using effect size calculations. Finally, a series of linear regression analyses were performed for various outcomes (eg, steps, minutes of MVPA, and sedentary time) with percent change as the dependent variable in each analysis. Predictors were group, study cohort, age, gender, BMI, previous pedometer use, and baseline value of the selected outcome variable. In addition, an interaction term between the group and baseline level of each outcome variable was included in each analysis; values for baseline activity levels were centered around the grand mean prior to calculation of this term. The alpha level was set at .05 for all analyses.

To evaluate the impact of habit formation on outcomes, we first compared group scores on the SRHI Automaticity Subscale for each of the 3 Fitbit-related habits using independent-sample *t* tests. For descriptive purposes, participants were also divided into those with high and low habit strength based on a median split of total Automaticity scores (across the 3 behaviors). Then, wear time (average valid wear days/week) was plotted over the course of the intervention. Finally, as individuals in both groups reported developing habits surrounding their Fitbits, correlation coefficients were calculated across all participants to examine associations among habit formation scores (eg, Automaticity scores for each of the 3 habits, including wearing the Fitbit, checking data on the Fitbit, checking data on the app, and the habit total score), FB usage averaged across the intervention period, for example, wear time and activity (eg, steps/day), and the active and sedentary behavior-related outcomes at follow-up.

## Results

Participants were primarily white, college-educated, and overweight. As shown in Table 1, groups were similar with respect to basic demographic characteristics and resting heart rate and blood pressure. In addition, groups were similar in their active and sedentary behaviors at the baseline, as shown in Table 2.

**Figure 2:**
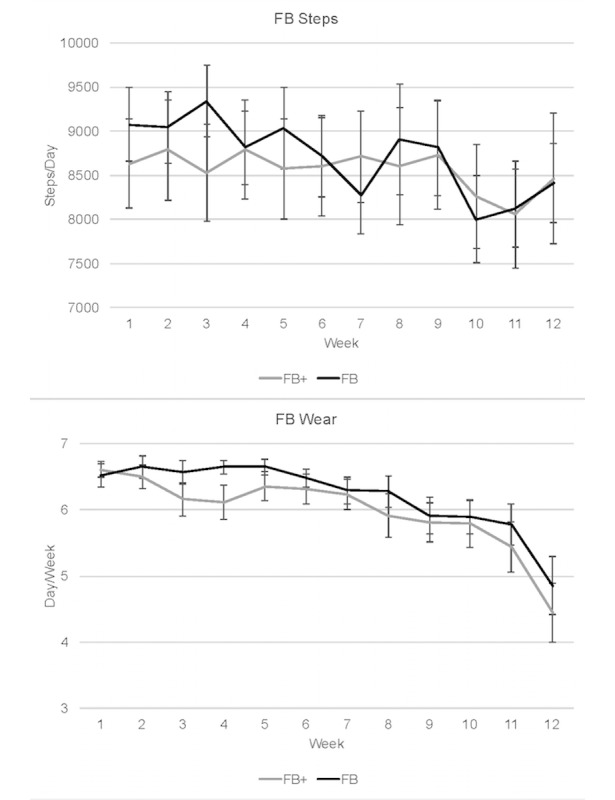
Wear data and steps collected from the Fitbit via Fitabase during the 12-week intervention. FB+: Fitbit plus motivational interviewing and habit education; FB: Fitbit alone.

Across the 12 weeks of the study, participants in both groups decreased the number of days/week the Fitbit was worn, with more notable declines in the final weeks (Figure 2). Similarly, even on days when the Fitbit was worn, steps/day during the intervention (measured via the Fitbit) decreased toward the end of the intervention period. There were no significant group differences at any time-point in any of these measures.

**Table 1:** Baseline participants’ characteristics.

Characteristics		Full sample (n=91)	Fitbit plus motivational interviewing and habit education (n=45)	Fitbit alone (n=46)	Group differences, *P* value
Age in years, mean (SD)		41.7 (9.3)	41.1 (9.2)	42.2 (9.4)	.56
Male, n (%)		43 (47)	20 (44)	23 (50)	.68
Education with college degree, n (% )		87 (97)	43 (96)	44 (96)	.83
Married, n (%)		73 (80)	36 (80)	37 (80)	.77
Income>50,000/year, n (%)		79 (87)	38 (84)	40 (87)	.69
White individuals, n (%)		72 (79)	33 (73)	39 (85)	.44
Employed full-time, n (%)		88 (97)	43 (96)	46 (100)	.34
Previous pedometer use reported as yes, n (%)		36 (40)	17 (38)	19 (41)	.83
Heart rate (bpm), mean (SD)		70.5 (11.6)	70.9 (11.9)	70.1 (9.1)	.74
**Blood pressure**					
	Systolic blood pressure (mm HG), mean (SD)	121.7 (16.5)	12.1 (17.5)	123.2 (15.6)	.37
	Diastolic blood pressure (mm HG), mean (SD)	76.5 (12.5)	74.9 (13.6)	78.1 (11.2)	.24
Height (cm), mean (SD)		171.9 (8.5)	17.9 (9.13)	172.8 (7.7)	.40
Weight (kg), mean (SD)		87.8 (21.4)	82.7 (21.4)	9.5 (18.9)	.21
Body mass index, mean (SD)		29.6 (6.3)	28.7 (6.3)	3.4 (6.2)	.20
% Body fat, mean (SD)		34.8 (8.2)	34.1 (9.0)	35.5 (8.6)	.43

As shown in Table 2, results comparing outcomes from pre to post demonstrated that participants in FB+ had small improvements in steps/day and MVPA in bouts of, at least, 10 minutes, while participants in FB had small decreases in steps per day and MVPA. For sedentary time, FB+ had small increases, and FB had small decreases. These changes were all nonsignificant. Effect size calculations comparing changes between groups showed that group differences in change over the intervention were also small in magnitude (*d*_range_=0.13-0.29). However, the effectiveness of the intervention was variable among participants with some individuals improving markedly and others decreasing activity levels over the 12-week period, as highlighted by the large SDs for change scores shown in Table 2.

**Figure 3:**
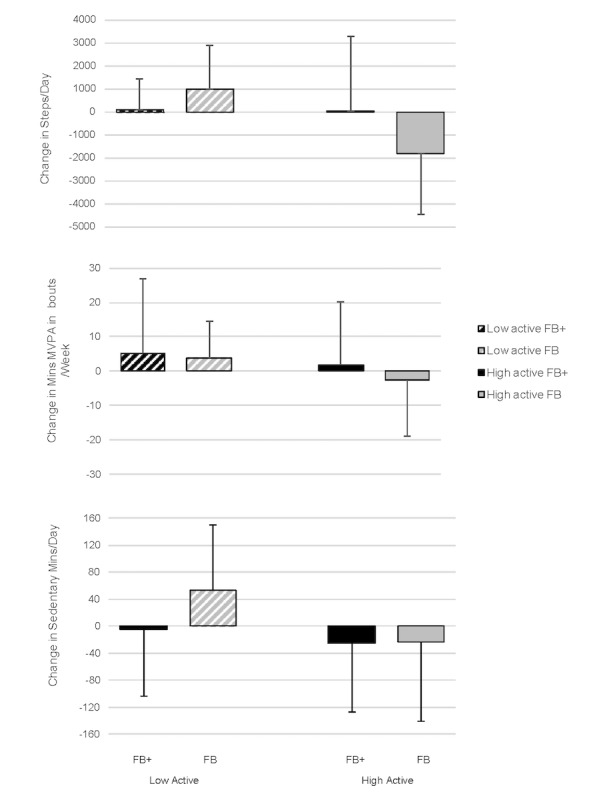
Within- and between-group comparisons of changes in active and sedentary behaviors measured via ActiGraph and activPAL pre-post based on original grouping (FB+, FB) and baseline activity level. Positive values for change scores indicate an increase from pre- to postintervention. FB+: Fitbit with motivational interviewing; FB: Fitbit alone.

This variability was partially explained when participants were further subdivided into high and low active using their baseline activity levels, as shown in Figure 3. Effect size calculations demonstrated that participants assigned to FB, who were low active at baseline (shown in the gray striped bars) had moderate improvements in both active and sedentary behaviors over time (*d*_range_=0.36-0.66). Those who were high active at baseline (shown in the solid gray bars), however, became less active and more sedentary across the 3 months (*d*_range_=0.18-0.95). A comparison of these changes within the FB group demonstrated that activity status at baseline had a moderate to large effect (*d*_range_=0.46-1.23) on the benefits of using FB. Differences within the FB+ group (shown in the solid and striped black bars) based on activity level at baseline were small in magnitude (*d*_range_=0.02-0.46) and generally favored the lower active group for variables related to PA and the higher active group for variables related to sedentary time.

Table 3 presents results from the linear regression analyses. Regressions demonstrated no significant difference in any of the primary outcomes (all *P*>.05) between the FB+ and FB groups. With respect to individual differences, lower baseline steps/day significantly predicted increases in daily steps over the intervention (*P*=.002). In addition, the interaction term (Group×Baseline steps; *P*<.001) was statistically significant. Specifically, as illustrated in Figure 3, participants with higher steps at baseline benefited more from being assigned to the FB+ group than the FB group. For changes in MVPA, significant predictors were previous experience with a pedometer (*P*=.03), and baseline minutes of MVPA (*P*=.01). Participants with no previous pedometer experience and lower levels of baseline MVPA showed greater improvements. For changes in sedentary time, the only significant predictor was baseline levels of sedentary time, such that participants with higher baseline levels of sedentary time had greater decreases in sedentary time postintervention (*P*=.003).

**Table 2:** Within- and between-groups comparisons of active and sedentary behaviors assessed using ActiGraph and activPAL monitors over the intervention.

Comparisons	Fitbit plus motivational interviewing and habit education				Fitbit alone				Between-groups effect size for change (Cohen *d*)
	Baseline, mean (SD)	12 weeks, mean (SD)	Change^a^ (post-pre), mean (SD)	Within-group effect size (Cohen *d*)	Baseline, mean (SD)	12 weeks, mean (SD)	Change^a^ (post-pre), mean (SD)	Within-group effect size (Cohen *d*)	
Average steps/day (n=91)	7496.88 (2895.94)	7574.38 (3499.38)	77.50 (2293.78)	.02	7519.60 (2259.13)	7221.80 (2106.18)	–297.80 (2658.84)	0.14	0.15
Average minutes of MVPA^b^/day (n=81)	68.57 (3.93)	68.91 (31.51)	.35 (3.96)	.13	75.06 (23.41)	69.93 (21.74)	–5.12 (24.40)	0.06	0.20
Average minutes of MVPA in 10+ min bouts/week (n=81)	77.99 (118.83)	93.16 (122.95)	15.17 (122.86)	.13	86.19 (78.99)	81.13 (77.55)	–5.07 (87.51)	0.17	0.19
Average minutes of sedentary time/day (n=91)	550.69 (102.74)	563.81 (106.53)	13.11 (99.68)	.14	547.96 (106.52)	529.94 (111.61)	–18.02 (112.82)	0.04	0.29
Average minutes of sedentary time in 30+ min bouts (n=91)	290.63 (128.34)	308.63 (127.37)	18.00 (88.41)	.01	279.35 (117.62)	284.22 (103.72)	4.87 (115.27)	0.23	0.13

^a^Positive values for change scores indicate an increase from pre- to postintervention.

^b^MVPA: moderate-to-vigorous intensity physical activity.

**Table 3:** Results from the regressions examining predictors of change in active and sedentary time measured using ActiGraph and activPAL monitors.

Independent variables	Dependent variables: percent change across the intervention							
	∆ Steps		∆ MVPA^a^			∆ Sedentary time		
	Standard beta	*P* value		Standard beta	*P* value		Standard beta	*P* value
Group	.01	.89		–.06	.57		–.15	.15
Cohort	–.15	.15		–.18	.10		.09	.34
Sex	–.01	.89		–.17	.11		–.14	.17
Age	.02	.83		.16	.15		–.03	.78
Body mass index	–.03	.76		–.04	.70		–.08	.41
Previous pedometer experience	–.13	.18		–.23	.03		–.02	.83
Baseline level of DV^b^ (steps, MVPA, sedentary time)	–.79	.002		–.49	.006		–.42	.003
Group×baseline level of DV	.49	<.001		–.04	.79		–.07	.61
Overall model	*R*^2^=0.27	<.001		*R*^2^=0.27	.003		*R*^2^=0.25	.003

^a^MVPA: moderate-to-vigorous intensity physical activity.

^b^DV: dependent variable.

**Table 4:** Descriptive statistics (mean [SD]) for the Automaticity Subscale of the Self-Reported Habit Index.

Self-reported habit index Automaticity Subscale	Full Sample (n=91)	Fitbit with motivational interviewing (n=45)	Fitbit alone (n=46)	Group differences (*P* value)
Wearing the Fitbit	16.3 (3.9)	16.7 (3.9)	15.8 (3.8)	.28
Checking data on the Fitbit	13.4 (4.8)	13.8 (5.2)	13.0 (4.4)	.44
Checking data using software or app	12.3 (4.9)	12.1 (5.1)	12.4 (4.8)	.78

**Figure 4:**
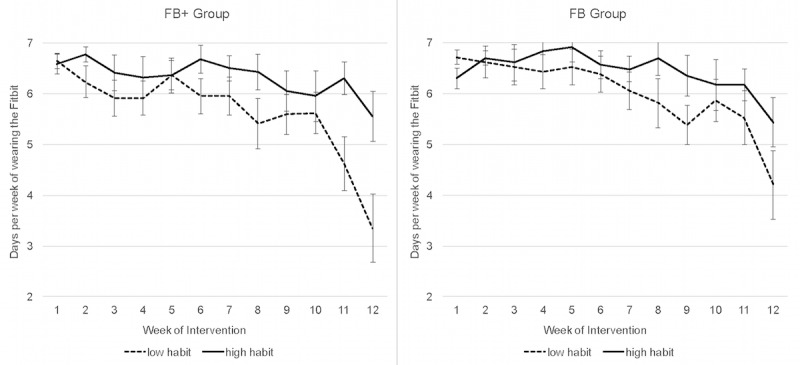
Fitbit wear time in relation to habit automaticity over the intervention period by the group (FB+, FB). High versus low habit was defined using a median split of the data. FB+: Fitbit with motivational interviewing; FB: Fitbit alone.

**Table 5:** The correlation matrix showing relationships among habits associated with wear and use of Fitbits, objectively measured Fitbit wear and steps during the intervention (data collected from Fitbits via Fitabase), and primary outcomes for active and sedentary behaviors (data collected via ActiGraph and activPAL monitors).

Habit and Outcome Variables	Habits				Fitbit (1-12 weeks), mean		MVPA^b^		Steps	Sedentary	
	Wearing FB^a^	Checking data on FB	Checking data on app	Total	Days of wear	Steps	Total	In bouts	Steps	Total	In bouts
Habits for wearing FB	1	N/A^c^	N/A	N/A	N/A	N/A	N/A	N/A	N/A	N/A	N/A
Habits for checking data on FB	.51^d^	1	N/A	N/A	N/A	N/A	N/A	N/A	N/A	N/A	N/A
Habits for checking data on app	.41^d^	.62^d^	1	N/A	N/A	N/A	N/A	N/A	N/A	N/A	N/A
Habit total	.74^d^	.88^d^	.84^d^	1	N/A	N/A	N/A	N/A	N/A	N/A	N/A
Days of FB wear (1-12 weeks), mean	.48^d^	.22^e^	.38^d^	.43^d^	1	N/A	N/A	N/A	N/A	N/A	N/A
FB steps (1-12 weeks), mean	.10	.27^e^	.35^d^	.30^d^	.21^e^	1	N/A	N/A	N/A	N/A	N/A
MVPA total	.02	.23^e^	.19	.18	.06	.78^d^	1	N/A	N/A	N/A	N/A
MVPA in bouts	.10	.21	.27^e^	.24^e^	.15	.67^d^	.86^d^	1	N/A	N/A	N/A
Steps	.02	.25^b^	.27^c^	.23^e^	.04	.69^d^	.85^d^	.71^e^	1	N/A	N/A
Sedentary total	.25^a^	−.06	−.11	.01	.10	−.40^d^	−.38^d^	−.24^e^	−.38^d^	1	N/A
Sedentary bouts	.11	−.18	−.17	−.12	.12	−.43^d^	−.47^d^	−.27^d^	−.47^d^	.83^d^	1

^a^FB: Fitbit.

^b^MVPA: moderate-to-vigorous intensity physical activity.

^c^N/A: not applicable.

^d^Significant at *P*<.01.

^e^Significant at *P*<.05.

Groups were not significantly different with respect to automaticity of habits surrounding the wearing and use of Fitbit, as shown in Table 4 (all *P*>.05). As shown in Figure 4, regardless of group, individuals reporting greater habit strength regarding Fitbit use were more likely to wear the Fitbit regularly during the second half of the intervention than those with lower habit strength. Results from the correlation analyses, shown in Table 5, demonstrated that habits surrounding the Fitbit were predictive of actual Fitbit wear and steps/day during the intervention (data from Fitabase). Postintervention, automaticity of habits surrounding checking the data on the Fitbit itself was most predictive of total minutes of MVPA and habit surrounding checking the data on the Fitbit App were most predictive of MVPA in bouts and average steps/day (data from ActiGraph and activPAL monitors). Correlations between Fitbit-related habits and sedentary time (total and in bouts) were largely small and nonsignificant, except habits regarding wearing the Fitbit being significantly associated with total minutes of sedentary time.

## Discussion

The primary purpose of this study was to evaluate the utility of a low-dose, MI-based health coaching intervention to enhance outcomes associated with the use of wearable activity trackers. The results demonstrated that the effects of using FB on PA behaviors in healthy adults were modest and highly variable. On average, for participants who received a Fitbit without additional support, PA levels declined slightly from pre- to postintervention. However, results showed that individuals who were less active at the baseline had moderate improvements in PA (eg, ~19% increase in steps/day); this is in contrast to those who were higher active at baseline who showed decreases of ~17% in both steps and minutes of MVPA/day over the 12 weeks. Thus, there may be some benefits to simply providing activity trackers to generally healthy adults who are insufficiently active. However, these results also highlight an important consideration in that active adults may not benefit from these devices without additional support.

In addition, these results demonstrated that the provision of low-dose health coaching had little effect on the sample as a whole. On average, participants receiving coaching increased their steps and minutes of MVPA, but the effects were small in magnitude. However, somewhat surprisingly, health coaching did provide an added benefit for those who were more active at the baseline. While active individuals who received a Fitbit without additional support had declines in their activity levels, those who received health coaching were able to maintain their high levels of activity throughout the intervention. Previous research has examined the effects of wearable technology in combination with a variety of other behavior change strategies, including education and goal setting, short message service text messages, and incentives. These studies found that the additional strategies were ineffective for improving outcomes associated with the provision of activity trackers [8-11].

In contrast to the additional behavior change strategies tested previously, the MI-based health coaching used in this study focused on the development of intrinsic motivation for behavior change, which should theoretically be more effective than strategies that focus on external motivators such as incentives [34]. Our results suggest that the health coaching did have benefits, but those were not equally distributed across participants. Future research examining different doses of coaching in concert with longer duration interventions will be critical for addressing these important questions.

This study adds to the growing body of research examining the effectiveness of wearable activity trackers and suggest that individual factors may influence the effectiveness of these devices for promoting increases in PA. Though there were several early studies showing that activity trackers had promise for increasing activity levels [35,36], there have also been larger trials that have failed to show that they significantly influenced behavior or health [10,37-39]. However, as described above, our data demonstrate that some individuals benefited a great deal from these devices, particularly those who were less active to start and, thus, potentially stood to gain the most from improved activity levels. Previous research in low-active samples is equivocal with respect to this finding with some evidence that activity trackers are beneficial [35] and other evidence that they do not substantively improve PA [39]. However, to the best of our knowledge, baseline activity levels have not been explicitly examined as a predictor of success with activity monitors. In addition, data from this study suggest that the lack of previous experience with a pedometer was also a potentially important predictor of improvements in PA. Thus, while these devices may not be effective for all individuals, they do appear to have substantial benefits for some. Additional research looking at individual differences in success with utilizing these devices is warranted to determine the profile of those who may benefit the most.

A possible explanation for the lack of impact in the studies noted above is the documented decline in interest and use of monitors over time after the novelty wears off. In line with this, investigations of wearable abandonment have shown that one-third of consumers discontinue the use of activity trackers after 6 months [40]; this is highlighted by a prominent and highly publicized study from Jakicic et al [37], which reported limited value from the inclusion of wearable activity trackers in standard weight loss programming. In this study, participants were provided with monitors for 18 months (~550 days), but the median number of days the monitor was worn was only 170, approximately 31% of available days, and the median wear time on the days the device was worn was 240 minutes out of a possible 1440 (~17% of the days). Similarly, in the Le et al study [39], monitors were only worn 19 days each month during the first 3 months of the intervention and usage declined to 15 days each month during the subsequent 3 months. Thus, the lack of impact in these and other studies may be attributed to reduced interest or lack of attention given to helping participants learn how to effectively use wearable monitors to promote sustained behavior change; our results reflected this as well. As highlighted in Figure 2, the number of days the Fitbits were worn each week declined from an average of 6.5-4.4 by the end of the intervention.

In addition, this study suggests that the habit development surrounding wear and regular interactions with these devices and their associated apps may be a key factor in promoting more sustained behavior change. Specifically, the habit development was predictive of wearing activity trackers more consistently during the final weeks of the intervention. Furthermore, habits, particularly those regarding the use of the Fitbit app, were predictive of greater levels of PA both during and following the intervention. Based on the design of the trial, it is difficult to determine whether habit developments played a causal role in behavior change. For example, it is possible that developing habits surrounding the use of the Fitbit led to improvements in PA. However, it is equally likely that those who were more successful at increasing PA were more likely to develop Fitbit-related habits. In addition, it should be noted that tying PA habits to an external device, like Fitbit, could be potentially problematic. These devices break, get lost, and people’s preferences for wearing them likely change over time. Moreover, the devices and associated apps evolve with some features being added or removed, altering the user experience. Thus, when using activity trackers to develop PA habits, the focus should be on establishing cues that are separate from the device itself to promote sustained improvements in PA [41].

To the best of our knowledge, the effects of activity trackers on sedentary behavior have not been examined. Although this study was not specifically targeted towards sedentary time, our results showed that in a group of generally healthy adults, this intervention had little effect on this set of behaviors, even in participants who improved their levels of PA; this is not surprising as previous research has demonstrated that interventions focusing on PA have little effect on the lower end of the intensity continuum [42]. Furthermore, the device used (Fitbit Charge) did not provide specific feedback on sedentary behaviors to the wearer. Sedentary behavior is an important contributor to health outcomes, and newer models of Fitbit and other similar devices do include features like idle alerts, which are designed to alert the wearer when they have been inactive for a prolonged period (eg, 50 minutes). Future studies using wearables with the intention of deceasing sedentary time should explicitly target sedentary behavior and include the use of a tracker that provides cues related to sedentary behavior and some level of feedback specific to these behaviors.

This study has a number of limitations. The sample was relatively small, generally healthy, and the duration of the trial was only 3 months. As such, the generalizability is limited to healthy adults and examination of factors predicting long-term adherence was not possible. Furthermore, we used a single, low-dose of health coaching; this was done purposely with an eye towards future translatability. However, it is possible that a higher dose of health coaching would have been more effective. Finally, our results demonstrating that lower active individuals improved more while individuals who were more active became less so could be interpreted as classic regression to the mean. However, the fact that higher active individuals receiving health coaching maintained their levels of activity across the intervention makes this a less likely explanation for these findings.

In summary, results from this study demonstrate that wearable activity trackers may have beneficial effects on PA in healthy adults. However, these benefits do not occur across the board and are more likely to be observed in individuals with lower levels of baseline activity. Furthermore, this study suggests that to engage more active individuals, additional behavior change strategies, like the provision of health coaching, may be needed. In addition, this study highlights the importance of habit development surrounding the wear and use of activity monitors and engagement with the associated software to promote increases in PA; this may be an important area to pursue, as making PA habitual is critical for realizing associated health benefits. As such, further research is warranted to determine whether explicit efforts to develop activity monitor-related habits can improve the effectiveness of these devices. Finally, additional research examining the effects of these devices alone or in combination with health coaching in subclinical and clinical populations is needed to determine whether they can be used in conjunction with more traditional strategies to prevent and treat chronic health conditions.

## Abbreviations

FB: Fitbit
FB+: Fitbit plus motivational interviewing and habit education
MI: motivational interviewing
MVPA: moderate-to-vigorous intensity physical activity
PA: physical activity
SRHI: Self-Reported Habit Index
